# Evidence of SARS‐CoV‐2 Exposure in Cats and Dogs From Households in Romania and Long‐Term Specific Seroconversion in Cats

**DOI:** 10.1002/vms3.70358

**Published:** 2025-04-28

**Authors:** Luanda Elena Oslobanu, Luciana Alexandra Crivei, Gheorghe Savuta, Laro Gómez‐Marcos, Pablo Nogales‐Altozano, José M. Rojas, Noemí Sevilla

**Affiliations:** ^1^ Faculty of Veterinary Medicine Regional Center of Advanced Research for Emerging Diseases Zoonoses and Food Safety “Ion Ionescu de la Brad” Iasi University of Life Sciences Iași Romania; ^2^ Centro de Investigación en Sanidad Animal Instituto Nacional de Investigación y Tecnología Agraria y Alimentaria Consejo Superior de Investigaciones Científicas (CISA‐INIA‐CSIC), Valdeolmos Madrid Spain

**Keywords:** zooanthroponotic transmission, long term seroconversion in cats, Romania, SARS‐CoV‐2 in pets, seroneutralisation

## Abstract

**Introduction:**

The COVID‐19 pandemic underscored the need to understand the zoonotic transmission of pathogens. SARS‐CoV‐2 has been reported to be transmitted from humans to pets, including cats and dogs, particularly after close contact with infected individuals. Studies have shown that cats are more susceptible to natural infection and can transmit the virus to other cats and humans. The zoonotic transmission route represents a risk for animal health workers. Despite global reports, data from Romania remain sparse. This study aimed to evaluate the presence of SARS‐CoV‐2 specific antibodies in household dogs and cats in Romania during the COVID‐19 pandemic.

**Methods:**

The study was conducted at a veterinary clinic in Iasi City, Romania, from March 2020 to December 2022. Blood samples were collected from 84 cats and 82 dogs that had been in contact with COVID‐19‐positive owners. Plasma samples were tested for anti‐SARS‐CoV‐2 antibodies using an ELISA test, followed by confirmation with a seroneutralisation (SN) assay. The SN assay used the SARS‐CoV‐2 MAD6 strain and Omicron strain to determine neutralising antibody titers.

**Results:**

ELISA testing showed a seroprevalence of 9.5% in cats and 11% in dogs. Further SN assay testing confirmed SARS‐CoV‐2 specific antibodies in 9.4% of cats and 2.4% of dogs. One cat maintained antibodies for over a year, though with reduced titers. Most cats' antibodies did not cross‐react with the Omicron strain, indicating limited cross‐reactivity. The study highlighted higher seroprevalence and antibody titers in cats compared to dogs, likely due to more efficient viral replication in cats.

**Conclusions:**

This study provides the first serological evidence of SARS‐CoV‐2 exposure in household pets during the pandemic in Romania, with findings suggesting cats are more susceptible to infection from their owners than dogs. The cats that were living in households in one area of the city were prone to be infected with SARS‐CoV‐2 from their owners and high levels of seroconversion were detected. These results align with global reports, demonstrating that cats are particularly susceptible to SARS‐CoV‐2 when living with infected owners.

## Graphical abstract

This study in Romania investigated SARS‐CoV‐2 antibodies in pets potentially exposed to infected owners during the COVID‐19 pandemic (2020–2022). The findings suggest that cats are more susceptible to SARS‐CoV‐2 infection than dogs, describe the long term persistence of antibodies in one cat, and asses the poor diagnostic value of a commercial ELISA kit. The results support global reports of pets being susceptible to SARS‐CoV‐2 infection when living close to infected individuals, providing one of the first pieces of evidence of SARS‐CoV‐2 exposure in pets in Romania, where data are scattered

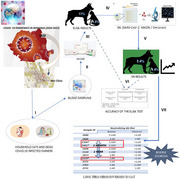

## 1 Introduction

The COVID‐19 pandemic brought into light the necessity to better understand the zoonotic aspects of different pathogens. SARS‐CoV‐2, responsible for the COVID‐19 pandemic in humans, has been reported to transmit from humans to dogs and cats in several instances. There have been cases of pets testing positive for the virus after close contact with infected individuals (Alberto‐Orlando et al. 2022). Most of the studies reported the detection of specific antibodies in sera or SARS‐CoV‐2 RNA in faecal or respiratory swabs collected from pets from epidemic areas (Abdel‐Moneim and Abdelwhab 2020). The SARS‐CoV‐2 variants identified in cats and dogs are genetically similar to the ones that were circulating in humans at the same time, thus suggesting that zooanthroponotic transmission is a common and ongoing occurrence (Galhardo et al. 2023). By now, it is known that cats have a higher susceptibility to natural infection in comparison with dogs (Halfmann et al. 2020; Gaudreault et al. 2020; Sila et al. 2022), and virus transmission to other cats both by direct and indirect contact in laboratory conditions has been demonstrated (Bosco‐Lauth 2020). In addition, studies managed to demonstrate the transmission of the virus from cats to humans (Sila et al. 2022). The epidemiologic impact of pet‐to‐human transmissions is likely low, but this route represents nonetheless a risk for animal health workers such as veterinarians (Nielsen et al. 2023). Therefore, evaluating the SARS‐CoV‐2 serological status of household pets could help estimate the likelihood of zooanthroponotic transmission, which ultimately could mitigate the risk of SARS‐CoV‐2 zoonotic retransmission.

Despite continuous reports of SARS‐CoV‐2 infections in pets worldwide, there are still scattered data from Romania regarding this occurrence (Morosan et al. 2023; Crețu et al. 2022). The first pandemic wave started in Romania in March 2020, followed by successive waves until February 2022 with the most important infection peaks occurring in spring and autumn. Studying the seroconversion of household pets to circulating human SARS‐CoV‐2 strains can help evaluate the probability of the virus transmission to pets. This study aimed to add data from Romania to the international research effort analysing the presence of specific antibodies to SARS‐CoV‐2 in household cats and dogs during the COVID‐19 pandemic in humans.

## 2 Materials and Methods

### 2.1 Location of the Study and Animal Sampling

The survey was conducted prospectively during healthcare visits of animals at a veterinary clinic in Eastern Romania. The choice of the clinic was random and was based on the availability of the sample bank. For the purpose of the study, blood samples collected from March 2020 to December 2022 from cats and dogs in a small animal private practice in Iasi City (47° 10′ 0.012″ N and 27° 36′ 0 E) which is the third largest urban area in the country, were tested. Data from the Local Centre of Public Health Iași showed that the COVID‐19 morbidity rates in the human population were 2.8% in 2020, 4.4% in 2021, and 5.5% in 2022 situating the area among the most affected counties affected by COVID pandemics in Romania (from a total of 42). As of November 2022, Iasi reported 125,727 people who tested positive for COVID‐19 being placed the 6th in Romania (Statista Research Department 2023), with an overall prevalence of 12.68% in the county.

A total of 166 plasma samples from 84 cats and 82 dogs (corresponding to 166 different animals and households), that had direct contact with owners diagnosed previously with COVID‐19 were tested. Information on each sampled animal was registered by the veterinarian. It included the signalment of the animal (species, breed, age, and gender, Table 1). Blood samples were collected via cephalic and saphenous venipuncture. The plasma was extracted and stored at −20°C until further use after collection. The records did not include the exact date of the owner's infection with SARS‐CoV‐2 but only that they were COVID‐positive in the last 6 months prior to the animal consultation. Authorised medical staff from the clinic performed the sampling during the diagnostic procedure with the owners’ permission. Informed consent was obtained from every participating owner to use samples for diagnosis purposes (haematology, serology, biochemistry). All the handling of the animals was done in the frame of the 205/2004 Romanian Animal Welfare Law.

**TABLE 1:** Information regarding the tested samples in cats and dogs.

CAT breed	No. of samples	Sex		Age category		
		F	M	Junior	Adult	Senior
Birman	2	1	1			2
Mixed breed	63	35	28	10	38	15
Siamese	11	9	2		5	6
Persian	6	1	5		2	4
Scottish	2	2	0	1	1	
Totals	**84**	48	36	11	46	27

DOG breed	No. of samples	Sex		Age category		
		F	M	Junior	Adult	Senior
Bichon	26	11	15		15	11
English Bulldog	2	1	1		2	0
German Shepherd	3	2	1		2	1
Canne Corso	1	1	0	1	0	0
Caniche	3	1	2		1	2
Chihuahua	1		1		1	
Cocker	2	1	1		2	
Mixed breed	12	11	1		6	6
Great Dane	1	1				1
Jack Russel	1		1		1	
Labrador Retriever	1		1		1	
Pekinez	13	8	5		8	7
Pincher	6	3	3		4	2
Pomeranian	1	1			1	
Pug	4	4		2	2	
Shih‐Tzu	2	2				2
Shar‐pei	1		1		1	
Teckel	1		1		1	
York Shire	1	1				1
Totals	**82**	48	34	3	48	33

### 2.2 Serology

All plasma samples were tested for the presence of anti‐SARS‐CoV2 antibodies by ELISA test (ID Screen SARS‐CoV‐2 Double Antigen Multi‐species) and were performed according to the manufacturer's instructions. Briefly, the double antigen ELISA targets antibodies against the nucleocapsid of the SARS‐CoV‐2 virus. Sample dilutions of 1:50 were used. Positive and negative controls were provided by the manufacturer. A purified N protein recombinant antigen horseradish peroxidase (HRP) conjugate was used. After washing, the TMB Substrate Solution was added. Using a microplate spectrophotometer (Epoch 2; Agilent BioTek), the absorbance values were recorded at 450 nm wavelength. The results were expressed as the percentage of inhibition (IP). Samples with a S/P ratio equal to or higher than 60% were considered positive, while if the IP was ≤ 50%, they were considered negative. Cat‐positive samples were tested twice by ELISA to verify the accuracy of the results. In addition, a cat was sampled and tested three times.

Both cat and dog‐positive samples by ELISA were tested by seroneutralisation (SN) assay. The SARS‐CoV‐2 MAD6 viral strain (genome sequence identical to the Wuhan‐Hu isolate, GenBank MN908947) and Omicron strain were used, which were the predominant circulating lineages in humans (2020/2022) in Romania. The variants circulating in Romania at the time of collection were assessed based on the results provided by the National Centre for Communicable Diseases Surveillance and Control Romania (Table 2), (Centrul Național de Supraveghere Şi Control al Bolilor Transmisibile—Analiză Cazuri Confirmate COVID19, n.d.). Serial dilutions of heat‐inactivated cat or dog sera (starting at 1:10) were incubated with 100 PFU of SARS‐CoV‐2 MAD6 or Omicron for 1 h at 37°C in 96‐well plates. Vero E6 cells of 2 × 10^4^ were then seeded on the sera/virus mixture and incubated for 3 days at 37°C, 5% CO_2_. Culture media was removed and cells were fixed with 2% paraformaldehyde prior to staining with 2% crystal violet. Neutralising antibodies (Nab) titers were calculated as the serum dilution at which less than 50% cytopathic effect was observed in replicate wells.

**TABLE 2:** SARS‐CoV2 variants circulating in Romania at the time of samples collection.

ID	Species	Sampling time	SARS Cov2 variants circulating in humans
*947*	dog	June 2020	Wuhan
1235	dog	October 2020	Wuhan
1326^a^	cat	November 2020	Wuhan
1421^a^	cat	December 2020	Wuhan
1426	cat	January 2021	Wuhan
1494	dog	January 2021	90%Wuhan/10% Alpha
1510	cat	February 2021	Wuhan / 30% Alpha
1604	dog	March 2021	80%Alpha/20%Omicron
1609	dog	March 2021	Alpha/ Beta
1613	cat	March 2021	95 %Alpha/Beta
1640	dog	April 2021	80%Alpha/20%Omicron
1680	dog	April 2021	75%Alpha/20%Omicron/
*1784*	dog	June 2021	Alpha 80% / 20%Delta
2034	dog	August 2021	Delta
2216	dog	November 2021	Delta
2259^a^	cat	November 2021	Delta
2271	cat	November 2021	Delta
2606	dog	June 2022	Omicron
2703	cat	August 2022	Omicron
2731	cat	August 2022	Omicron
2776	cat	September 2022	Omicron
*2882*	dog	November 2022	Omicron
2884	cat	November 2022	Omicron
*3081*	cat	February 2023	Omicron

^a^ tested in triplicate.

### 2.3 Statistical Analyses

Results from the serology analyses, registered information per individual cat and dog, were entered into a Microsoft Excel spreadsheet. The seroprevalence was calculated at a 95% confidence interval, based on the binomial distribution, using the web‐based Free Statistics Calculators (Free Statistics Calculators, n.d.). The sensitivity and specificity of the ELISA against the SN test were calculated using the basic formulas as described by Trevethan R., (Trevethan 2017), where the golden standards were considered the SN test results.

## 3 Results

To investigate the prevalence of SARS‐CoV‐2 in domestic cats and dogs living in close contact with owners, plasma samples were collected from animals living in a district of Iasi City, Romania. SARS‐CoV‐2 ELISA showed a **9.5%** seroprevalence in cats (8/84), [CI 95%: 3.25–15.8] and 11% seroprevalence in dogs (9/82), [CI 95%: 4.21–17.74] in plasma samples (Table 3).

**TABLE 3:** Comparative results for the detection of specific anti COV 2 Ab with ELISA and SNT.

Sample ID	Breed/age/sex	ELISA result	Neutralizing Ab titer	
			Wuhan	Omicron
947	Bichon/12yo/M	N	<1:20	<1:20
1235	Pincher/7 yo/F	*P*	<1:20	<1:20
1494	Bichon/13 yo/F	*P*	<1:20	<1:20
1604	Shar‐pei/ 6 yo/M	*P*	<1:20	<1:20
1609	Bichon/17 yo/F	*P*	<1:20	<1:20
1640	Mixed breed/3 yo/F	*P*	<1:20	<1:20
1680	**Great Dane/10 yo/F**	**P**	**1:140**	<1:20
1784	Bichon/6 yo/F	*P*	<1:20	<1:20
2034	**Bichon/9yo/F**	**N**	<1:20	**1:20**
2216	Pekinez/12 yo/F	*P*	<1:20	<1:20
2606	**Chihuahua/1.5 yo/M**	**P**	**1:20**	**1:80**
2882	Pekinez/12 yo/M	**N**	<1:20	<1:20
2776	Mixed breed/6yo/F	D	<1:20	<1:20
3081	Mixed breed/ 7 yo/M	N	<1:20	<1:20
1326*	**Mixed breed/13yo/M**	**P**	**1:320**	<1:20
1421*	**Mixed breed/13yo/M**	**P**	**1:320**	<1:20
1426	**Mixed breed/0.75 yo/F**	**P**	**1:320**	<1:20
1510	**Mixed breed/3.5 yo/M**	**P**	**1:160**	<1:20
1613	**Mixed breed/2 yo/F**	**P**	**1:80**	<1:20
2259*	**Mixed breed/14yo/M**	**P**	**1:80**	<1:20
2271	**Mixed breed/7 yo/F**	**P**	**1:20**	<1:20
2703	**Mixed breed/1.5 yo/M**	**P**	**1:80**	**1:160**
2731	**Mixed breed/2 yo/M**	**P**	**1:40**	**1:20**
2884	**Mixed breed/15.5 yo/M**	*P*	<1:20	<1:20

*Note*: N‐negative and P‐positive samples tested by ELISA.

Abbreviations: F, female; M, male.

To further confirm the presence of SARS‐CoV‐2 specific antibodies, the positive sera were tested by SN assay. Out of the eight positive samples detected by ELISA in cats, seven were also positive **(9.4%)** [CI 95%: 3.2–15.6] in SN assays (SN titers ranging from 1:20 to 1:320) whereas from the nine positive dog samples, Nab were detected in only two samples **(2.4%)** [CI 95%: ‐0.9–5.78] (SN titers ranging from 1:20 to 1:80). The negative samples in ELISA were also negative in SN assays (Table 3). The overall sensitivity of ELISA calculated against the SN test was 60% and the specificity was 45.71%.

Most NAb in cats blocked infection with the Wuhan MAD6 strain but not the Omicron strain, which suggests that pet infection did not generate cross‐reactive NAb. One dog S2606 possessed NAb to both Wuhan and Omicron strains (Table 3) suggesting a coinfection. In cats, there were two samples (S2703, S2731) in which NAb to both lineages were detected. Regarding clinical signs, the majority of the animals presented to the veterinarian for routine checks or surgical interventions. However, S2731 suffered from acute gastroenteritis (that was treated successfully in the clinic, and cat S2703 had a febrile syndrome and a regenerative anaemia.

Re‐testing of one cat that was in the evidence of the clinic with liver failure and was monitored after one year showed persistence of circulating antibodies with a 4‐fold reduction of the NAb titer from 1: 320 to 1:80 (samples no. 1326 and 2259), but with the same neutralisation level when tested at a month interval (sample no. 1326 and 1421). No data about the owner's COVID‐19 infection were gathered after the first sampling in December 2020.

## 4 Discussion

Our survey showed similar results to studies found in other countries in animals that were only housed indoors or had also street access (Table 4). Higher levels of neutralizing antibodies against SARS‐CoV‐2 were found in cats and low levels of antibodies and a lower seroprevalence in dogs (Gaudreault et al. 2020). Previous studies also showed that cats owned by COVID‐19 patients are more prone to develop higher neutralisation titers due to the close contact between cats and COVID‐19 patients (Zhang et al. 2020).

**TABLE 4:** Literature review regarding **SARSCoV‐2** seroprevalence in pets (ELISA and SN^*^).

	No. of tested samples		SARSCoV‐2 Prevalence (%)		
Country	cats	dogs	cats	dogs	References
Brazil	182	762	2.2	4.3	Jarrah et al. 2023
Swiss	172	49	4.65	4.08	Kuhlmeier et al. 2023
Wuhan	102	—	10.8	—	Zhang et al. 2020
USA	956	1336	0.4	0^*^	Barua et al. 2021
The Netherlands	240	—	0.8^*^	—	van der Leij et al. 2021
France	143	165	8.4^*^	5.4^*^	Bessière et al. 2022
Italy	191	451	5.8^*^	3.3^*^	Patterson et al. 2020
Spain	114	—	3.51	—	Villanueva et al. 2022
Portugal	69	148	21.74	4.73	Barroso et al. 2022

* Tested by SN.

Although, seroconversion in household cats and dogs was similar in our study using the ELISA test, the presence of Nab was markedly higher in cats, both in terms of percentage and antibody titers. SARS‐CoV‐2 replication is more efficient in cats than in dogs (Shi et al. 2020). This could lead to increased antigen exposure in cats, which would prompt potent adaptive immune responses characterised by Nab. In dogs, viral replication is poor, the infection is likely controlled mainly through innate immune mechanisms, resulting in lower activation of adaptive immune mechanisms such as the induction of Nab (Nielsen et al. 2023; Tomeo‐Martín et al. 2024). It should be noted that in the absence of the exact infection time of the pet owner with SARS‐CoV‐2, some of these differences could be attributed to sampling in dogs at later time points in which antibody titers have subsided, although this seems unlikely given the higher titer of Nab consistently detected in cats in our study. Nonetheless, it should be noted that one limitation of this study is the fact that the information about the occurrence of SARS‐CoV‐2 infection in owners was limited to the owners reporting it within six months prior to the sampling The seroprevalence we report herein could be lower than the real infection burden in pets, due to antibody titers that could have declined below the detection threshold of our techniques within the testing timeframe that we had. Our data also show that little cross‐reaction exists between MAD6‐specific Nab and Omicron antibodies in cats. This is in line with studies in humans that have found that infection with the Wuhan strain conferred little to no cross‐reactive Nab to omicron strains. Conversely, infection with omicron also provided a limited generation of Nab to Wuhan strains in unvaccinated individuals (Richardson et al. 2022). Thus, it is possible that in the cases of the dog and cats that presented antibodies to both Wuhan and Omicron SARS‐CoV‐2 lineages, repeated infection of these animals has occurred, first with Wuhan‐related strains and then with an Omicron‐related virus. In the absence of longitudinal samples, it is however impossible to confirm this, since it could also be the result of the generation of cross‐reactive Nab in these animals.

The reported prevalences differ from one study to another and may be related to the heterogeneity of the data taken into account (e.g. different animal populations, different test characteristics, and the characteristics of the pandemics at the time of sampling). The slightly higher seroprevalence in cats in our study compared to studies in other countries (Table 4), can be explained by the origin of the pets that were living inside the house in close contact with the owners. Studies reporting a lower seroprevalence took into consideration samples from stray or hospitalised cats (Brazil 2023), or only from stray cats (Spain 2021). Other studies targeting households’ animals reported seroprevalence varied from 0.2% in Dutch dogs without known contact with COVID‐19‐positive persons (Zhao et al. 2021) to 53% in dogs living in COVID‐19‐positive households in France (Fritz et al. 2021). In Italy, 3.3% of dogs and 5.8% of cats presented specific antibody responses (Patterson et al. 2020).

The findings about the persistence of specific seroneutralising antibodies in the present work are different from a previous study in which persistence was limited to 110 days (Zhang et al. 2020) since we still detected an NAb titer of 1:80 after one year in one cat. This is in accordance with more recent studies that showed a longer persistence of neutralising antibodies in pets (Decaro et al. 2022).

The use of ELISA against the nucleocapsid protein of SARS‐CoV‐2 that was commercially available at the time of starting our study was one of the study limitations. The overall sensitivity of ELISA calculated against the SN test was 60% and the specificity was 45.71%. Most Nab target the spike protein, thus our ELISA based on detecting antibodies to the nucleocapsid is unlikely to be a good predictor of neutralizing antibodies. Indeed, a comprehensive study comparing the use of different serological methods showed that spike protein‐based ELISAs are the most accurate tests for serodiagnosis of SARS‐CoV‐2 infections in cats and dogs (Diezma‐Díaz et al. 2023). Our data regarding the seroconversion are also in line with those results based on the comparison between anti‐nucleocapsid ELISA and SN test in dogs. It is likely that this difference is less notable in cats because viral replication is more efficient in this species (Shi et al. 2020). Increased replication would result in more antigen availability in cats, which in turn would induce higher antibody titers, both to the nucleocapsid and the spike protein, thus increasing the likelihood of neutralising antibody generation. However, serological testing using ELISA is the preferred choice in screening and preliminary seroconversion studies due to its high throughput, cost‐efficiency, safety, automation, and rapid turnaround time. For monitoring and surveillance of pathogens with a zoonotic potential such as SARS‐CoV‐2, the use of accurate ELISA kits for preliminary studies is highly recommended.

## 5 Conclusion

This is the first study that provides reliable serological evidence of SARS‐CoV‐2 exposure in household pets during the pandemic in Romania and describes the persistence of antibodies in one cat proving a long term seroconversion. The cats that were living in households in one area of the city were prone to be infected with SARS‐CoV‐2 from their owners and, high levels of seroconversion were detected. The ELISA commercially available at the time of testing and used in the study showed a low sensitivity in comparison with the SN assay. Based on our results, we recommend the use of a test with a higher sensitivity for serological screening as suggested also by other studies. The results of the seroconversion study from Romania, are in concordance with previous serological studies made in households with SARS‐CoV‐2 infected individuals that were cohabitating with their pets during the COVID‐19 epidemics. We are confirming that dogs and cats are susceptible to the infection, with cats being more susceptible in comparison to dogs.

## Author Contributions

**Luanda Elena Oslobanu**: Conceptualisation, data curation, formal analysis, investigation, methodology, resources, writing – original draft. **Luciana Alexandra Crivei**: Formal analysis, investigation. **Gheorghe Savuta**: Formal analysis, project administration, supervision, visualisation. **Laro Gómez‐Marcos**: Data curation, formal analysis, investigation, methodology, writing – original draft. **Pablo Nogales‐Altozano**: Investigation, methodology, validation. **José M. Rojas**: Conceptualisation, data curation, resources, validation, writing – original draft, writing – review and editing. **Noemí Sevilla**: Conceptualisation, formal analysis, funding acquisition, investigation, methodology, visualisation, writing – review and editing.

## Ethics Statement

The authors confirm that the ethical policies of the journal, as noted on the journal's author guidelines page, have been adhered to. No ethical approval was required as this is a serological investigation using samples from a sample data base. Authorised medical staff performed the sampling during the diagnostic procedure with the owners’ permission. Informed consent was obtained from every participating owner to use samples for diagnosis purposes (haematology, serology, biochemistry). All the handling of the animals was done in the frame of the 205/2004 Romanian Animal Welfare Law.

## Conflicts of Interest

The authors declare no conflicts of interest.

### Peer Review

The peer review history for this article is available at https://www.webofscience.com/api/gateway/wos/peer‐review/10.1002/vms3.70358.

## Data Availability

All data generated or analysed during this study are included in this article.
